# From ensembles to meta-ensembles: Specific reward encoding by correlated network activity

**DOI:** 10.3389/fnbeh.2022.977474

**Published:** 2022-09-08

**Authors:** Christoph Körber, Wolfgang H. Sommer

**Affiliations:** ^1^Department of Functional Neuroanatomy, Institute of Anatomy and Cell Biology, Heidelberg University, Heidelberg, Germany; ^2^Medical Faculty Mannheim, Institute of Psychopharmacology, Central Institute of Mental Health, Heidelberg University, Mannheim, Germany

**Keywords:** alcohol, substance abuse, reward-seeking, neuronal ensemble, graph theory, infralimbic, amygdala, insula

## Abstract

Neuronal ensembles are local, sparsely distributed populations of neurons that are reliably re-activated by a specific stimulus, context or task. Such discrete cell populations can be defined either functionally, by electrophysiological recordings or *in vivo* calcium imaging, or anatomically, using the expression of markers such as the immediate early gene cFos. A typical example of tasks that involve the formation of neuronal ensembles is reward learning, such as the cue-reward pairing during operant conditioning. These ensembles are re-activated during cue-presentation and increasing evidence suggests that this re-activation is the neurophysiological basis for the execution of reward-seeking behavior. Whilst the pursuit of rewards is a common daily activity, it is also related to the consumption of drugs, such as alcohol, and may result in problematic behaviors including addiction. Recent research has identified neuronal ensembles in several reward-related brain regions that control distinct aspects of a conditioned response, e.g., contextual information about the availability of a specific reward or the actions needed to retrieve this reward under the given circumstances. Here, we review studies using the activity marker cFos to identify and characterize neuronal ensembles related to alcohol and non-drug rewards with a special emphasis on the discrimination between different rewards by meta-ensembles, i.e., by dynamic co-activation of multiple ensembles across different brain areas.

## Introduction

Survival in a changing environment requires experience-dependent or associative learning. This mental process can be empirically studied by the pursuit of consummatory rewards such as food or water. Pleasurable feelings generated by reward consumption act as a positive reinforcement, thereby motivating us to obtain this reward again (McClure et al., 2004). However, powerful rewards can also be obtained from drugs (Kelley and Berridge, 2002). Importantly, the value of any reward depends on the context and the current needs of an individual. A stream’s burbling may serve as a cue for a drink to a thirsty person, for cooling on a hot day or have no reward-association at all when hiking through the rain. Accordingly, the behavior required to obtain a reward, commonly referred to as reward-seeking, depends on the context of stimulus/cue presentation and the recall of a previously established cue-reward memory. Such memories can persist for long times and in the case of drugs, can elicit excessive seeking behavior and relapse even after prolonged periods of abstinence.

The drug with arguably the highest health burden is alcohol (WHO, 2018), which is consumed in moderate quantities by most of the world’s population but excessively by a minority which may develop alcohol use disorder (AUD). A phenomenon often reported by individuals with problematic consumption habits are intrusive thoughts or urges, also called craving. In AUD patients, craving can strongly diminish control over consumption, biased choice and relapse into excessive drinking (Heilig et al., 2019). Thus, gaining insights into the neuronal mechanisms of memory formation, decision making and seeking behavior for drug and natural rewards may open new roads for regaining control in AUD and related diseases (Heinz et al., 2020).

The processing of reward memories and the control over seeking behavior is thought to involve activation of the brain’s reward system, mesolimbic circuits originating in the ventral tegmental area and connecting striatal and prefrontocortical regions as well as the amygdala (Fein and Cardenas, 2015; Lüscher, 2016). These brain regions are broadly activated by reward consumption as well as the presentation of reward-associated cues, leading to the hypothesis that drugs hijack a circuitry that normally serves reward-related learning (Wise, 1987; Nesse and Berridge, 1997).

However, recent studies suggest that presentation of reward cues only activates small subsets of sparsely distributed neurons in a given region (∼3–15%; e.g., Koya et al., 2009; Bossert et al., 2011; Pfarr et al., 2015), which are conceptualized as neuronal ensembles (Hebb, 1949), implying that their activation controls reward-seeking behavior. In this review, we discuss recent progress concerning the importance of neuronal ensembles in reward-seeking. We pay particular attention to recent application of graph theory-based network analysis and the role of co-activation patterns of neuronal ensembles across multiple brain regions with regard to seeking behavior for alcohol and natural, sweet rewards.

## Neuronal ensembles in reward-seeking

Neuronal ensembles are defined as assemblies of neurons that are specifically and reliably activated by a stimulus (Hebb, 1949), although the exact form of co-activation of neurons within an ensemble can range from synchronous action potential firing to synfire chains to activation at some time during a behavioral task (Russo and Durstewitz, 2017). Such a wide definition allows for a variety of methodological approaches to identify the neurons participating in an ensemble and to monitor their activity. High resolution activity patterns in the millisecond range can be obtained by *in vivo* electrophysiology (e.g., Emberly and Seamans, 2020; Takehara-Nishiuchi et al., 2020; Sachuriga et al., 2021) or calcium imaging (e.g., Shin et al., 2020; Grant et al., 2021) and early studies using these techniques already suggested the existence of different ensembles involved in cocaine and sugar seeking within the nucleus accumbens (Carelli and Wightman, 2004). However, the application of these methods is typically restricted to one brain region and therefore, of limited use for investigating the distribution and interaction of local ensembles across the brain. Furthermore, the real-time detection of the sparsely distributed neurons participating in an ensemble is challenging due to the probabilistic association of environmental stimuli, neuronal responses and behavioral output. Alternatively, ensembles can be defined by the expression of activity markers, such as cFos, NF-κB, or Arc. These markers efficiently label recently activated neurons during a behavioral experiment and provide excellent, single neuron resolution across the entire brain.

The majority of studies relevant for this review have employed the immediate early gene and transcription factor cFos, an effector linking gene expression to synaptic plasticity in a position- and time-dependent manner (Morgan and Curran, 1989). Because of its rapid response properties and short half-life, it is particularly suited to monitor neuronal activity within a certain time window, i.e., stimulus presentation (Kaczmarek, 1993). cFos expression has been directly linked to distinct cellular, neurochemical and behavioral responses (Sommer et al., 1993; Sommer and Fuxe, 1997), and more recently to cue-induced drug or natural reward-seeking (Cruz et al., 2013; Pfarr et al., 2015). Interference with cued ensemble activation can specifically alter cue-triggered behavior, suggesting that neuronal ensembles underly the formation of memory engrams (Josselyn and Tonegawa, 2020). Hence, a local neuronal ensemble formed during the initial learning of a cue-reward association is reactivated upon re-exposure to the cue, which in turn leads to the recall of the reward memory and reward-seeking behavior (e.g., Bossert et al., 2011; Pfarr et al., 2015).

Neuronal ensembles as the basis for reward-seeking have received increasing attention in the past decade, as classical experiments, using lesions or gross inhibition of whole brain regions, produced conflicting results (Cruz et al., 2013). The indiscriminate inactivation of both, the sparse cue-specific ensemble and the vast majority of unrelated neuronal populations may obscure the function of a region. Thus, a brain region’s function during a task may be dominated by a distinct ensemble, or this ensemble’s role is hidden in a high tonic activity. In recent years, causality between the activation of cue-associated neuronal ensembles reward-seeking has been elegantly demonstrated using the Daun02-inactivation method (Koya et al., 2009; Cruz et al., 2013). This approach relies on transgenic *cfos-lac*Z rats (Kasof et al., 1995) which express the bacterial enzyme β-galactosidase under the *cfos* promoter. Neurons strongly activated during a behavioral task express cFos and consequently also β-galactosidase. This enzyme converts the prodrug Daun02 into the neurotoxin daunorubicin. Hence, local administration of Daun02 causes apoptosis of previously activated cells, but spares other neurons (Farquhar et al., 2002; Pfarr et al., 2015). Thereby, cFos-defined ensembles can be directly linked to distinct aspects of reward-seeking. Considering alcohol, seeking-related ensembles have been reported in the infralimbic (IL) cortex (Pfarr et al., 2015; Laque et al., 2019), nucleus accumbens (Leão et al., 2015) and amygdala (de Guglielmo et al., 2016). Another alcohol reward-related ensemble in the accumbens was found using NF-κB (nuclear factor kappa-light chain of activated B cells) as an activity marker and driver of the *lacZ* transgene (Nennig et al., 2018). Interestingly, in this study only about 60% of the induced NF-κB^+^ cells were neurons, suggesting glia cells as potential ensemble participants. Activity triggered ablation of a specific cue-associated ensemble in the IL, but not inactivation of the entire region, led to excessive seeking behavior (Pfarr et al., 2015), demonstrating that a small population of IL neurons (∼12%) was responsible for suppressing cue-induced alcohol-seeking. The same ensemble was not involved in stress-induced alcohol-seeking. Thus, specific manipulation of a reward-associated neuronal ensemble strongly implicated a group of neurons in the IL as the cellular correlate of a memory that controls specific aspects of reward-seeking, whereas an ensemble in the neighboring prelimbic region active during the same task had no effect on the behavioral output in this paradigm. Using the Daun02 chemogenetic approach, Suto et al. (2016) demonstrated coexisting neuronal ensembles within the IL that are selectively reactive to different environmental cues and either promoted or suppressed sweet reward-seeking. This extended findings of intermingling cFos ensembles mediating both, food reward and extinction memories in the IL (Warren et al., 2016). Furthermore, context-dependent roles of a sweet reward-seeking related ensemble for distinct aspects of behavioral expression were identified by cFos tagging experiments in mice (Jessen et al., 2022).

Taken together, using neuronal activity markers allows the detection of multiple, context/cue-specific ensembles coexisting within the same mPFC region that mediate different and even opposing aspects of behavior. This conclusion is also supported by *in vivo* electrophysiology and calcium imaging studies (Moorman and Aston-Jones, 2015; Otis et al., 2017). Importantly, ensembles involved in the control of reward-seeking are neither limited to the mPFC nor alcohol and sweet rewards, but have been found in various regions of the brain and for most known rewards (e.g., Bossert et al., 2011; Cruz et al., 2014; Leão et al., 2015; Rubio et al., 2015; de Guglielmo et al., 2016; Warren et al., 2016, 2019; Laque et al., 2019; Sieburg et al., 2019; Kimbrough et al., 2021). Thus, reward memories are represented in neuronal ensembles dispersed across the brain with ensembles in various regions involved in the control of seeking behavior. Moreover, different aspects of behavioral control (e.g., approach or avoidance) can be supported by ensembles within the same brain region, indicating major influence of experimental conditions on behavioral outcome, rather than clear evidence for functional segregation between brain regions.

## Neuronal ensembles for different rewards can be highly similar

**FIGURE 1:**
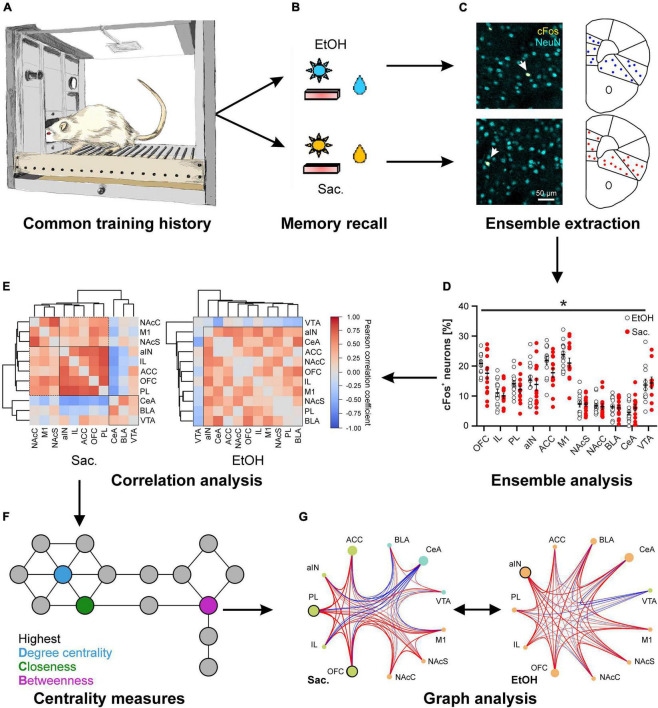
Distinct cue-reward memories are encoded by specific meta-ensembles consisting of co-active neuronal ensembles spread across multiple brain regions. Rats trained on a concurrent 2-reward operant self-administration paradigm establish memories for both rewards **(A)** that are retrieved under reinstatement conditions, meaning that previously learned reward-specific cues are presented but seeking behavior is not rewarded **(B)**. **(C,D)** Postmortem cFos expression analysis allows the analysis of neuronal ensembles within distinct brain areas. **(C)** Representative images of immuno double-labeling of cFos and the pan-neuronal marker NeuN, to determine the fraction of activated neurons, in the central amygdala after reinstatement for alcohol (top) and saccharin (bottom). **(D)** Quantification of the fraction of cFos labeled, activated neurons in 10 different reward-related brain regions after reinstatement for either alcohol (EtOH, white circles) or saccharin (Sac., red circles) (Data is presented as mean ± s.e.m., *p < 0.05, two-way ANOVA, main effect of group). **(E–G)** Reward-specific properties of brain activity are only evident after analysis of co-activity across brain regions **(E)** and subsequent graph theory-based analyses **(G)** such as analyses of centrality measures **(F)**. **(E)** Hierarchical clustering of brain regions according to the Pearson correlation coefficients derived from the fraction of cFos labeled neurons after reinstatement for saccharin (Sac., left) or alcohol (EtOH, right). **(F)** Scheme illustrating three commonly used centrality measures to analyze networks. The degree centrality is defined as the number of nodes directly connected to the node of interest. The closeness and betweenness are measures for the distance from the node of interest to all other nodes and the number of shortest connections between all other nodes that run *via* the node of interest, respectively. That means, nodes with a high degree centrality have many direct neighbors while those with a high closeness are at short distance to many other nodes. A high betweenness on the other hand indicates that a lot of (short) connections between other nodes, and thus information, run *via* the node of interest. **(G)** Example for the analysis of networks derived from the fractions of cFos labeled neurons after reinstatement for saccharin (Sac., left) and alcohol (EtOH, right) showing the recruited meta-ensembles. The size of the circles represents the importance of the brain region in information flow derived from centrality analysis and the strength and color of the connecting lines represents properties of network edges based on the Pearson correlation coefficients (thicker lines = stronger correlation, red: positive correlation, blue: negative correlation). Brain region labels: ACC, anterior cingulate cortex; aIN, anterior insular cortex; BLA, basolateral amygdala; CeA, central amygdala; IL, infralimbic cortex; M1, primary motor cortex; NAcC, nucleus accumbens core; NAcS, nucleus accumbens shell; OFC, orbitofrontal cortex; PL, prelimbic cortex; VTA, ventral tegmental area. [cFos staining in panel **(C)** as well as panels **(D–G)** reproduced from Wandres et al. (2021)].

An important question for a better understanding of reward-seeking and associated aspects of AUD is how memories of different rewards are represented in the brain. And along the same lines, whether there are distinct differences in the representation of natural and drug rewards that can explain the different behavioral outcomes, i.e., the bias toward excessive or compulsive responding that is characteristic for addiction. As outlined above, neuronal ensembles associated with seeking behavior for both reward types can be present within the same brain region, a finding that is also supported by *in vivo* functional magnetic resonance imaging (MRI) studies in humans and animals (Dudek et al., 2015; Noori et al., 2016). Recently, we addressed the identity of neurons comprising neuronal ensembles associated with alcohol (drug) and saccharin (natural reward) by employing a concurrent 2-reward operant self-administration paradigm in which rats learned to associate specific conditioned cues (odors) with the availability of a paired reward (alcohol or saccharin) and to execute the appropriate behavior in order to obtain the reward (press the correct lever). This experimental protocol ensured that each animal had the same training history and consequently had formed neuronal ensembles for both cue-reward pairs. Using event-specific labeling of cFos expression by two-color fluorescence *in situ* hybridization (FISH), we identified neurons that were active during the presentation of either one of the paired cues or during both cue presentations, in successive sessions under reinstatement conditions. Taking advantage of the rapid splicing of *cfos*-mRNA (Kasof et al., 1995), the mature mRNA resulting from the first session could be readily distinguished from the nascent *cfos*-mRNA present directly after the second session. Interestingly, within the IL both ensembles largely overlapped and only about 25% of *cfos*-mRNA positive neurons could be specifically attributed to either reward with no differences in cortical layer distribution (Pfarr et al., 2018). Thus, each reward seems to recruit a specific subset of neurons in the IL that signal the availability of the specific paired reward, while another subset of neurons may be recruited by rewards in general. The overall size of the alcohol or saccharine associated ensembles appeared to be quite similar in all brain regions examined and seemed to primarily depend on the brain region and not on the specific reward (Figure 1; Pfarr et al., 2018; Wandres et al., 2021). Likewise, neuronal ensembles in the dorsomedial shell of the NAc, activated by two stimuli of the same valence (morphine and cocaine) have been found to be highly overlapping, too, while stimuli of opposing valences (morphine and foot shock) activated ensembles that showed limited overlap (Xiu et al., 2014; Nawarawong and Olsen, 2020). However, studies in the NAc core and the ventromedial PFC using sucrose and cocaine as concurrent rewards (same valence) found less overlapping neuronal ensembles (Bobadilla et al., 2020; Kane et al., 2021).

Taken together, the current data suggest that the differences in encoding reward memories for drugs and natural rewards do not necessarily lay in the size or location of the neuronal ensemble, although ensembles in distinct brain regions such as the IL may control certain aspects of reward-seeking behavior and are thus critical to understanding the neurobehavioral processes underlying regaining control over drug taking and seeking.

## Different rewards are represented by meta-ensembles

To gain insight into the differences in memory representation of drug and natural rewards, so far, most studies on the neuronal correlates of reward-seeking focused on one or only a handful of brain regions, ignoring the complexity of the brain’s reward system and possible interactions between neuronal activation patterns in different brain regions. We, therefore, investigated cFos patterns across the reward system in animals that had been trained on the concurrent 2-reward operant self-administration paradigm (Pfarr et al., 2018; Wandres et al., 2021). Since we found no evidence for reward specific encoding by any of the analyzed regions (Figure 1D), we turned to graph theory-based network science, an approach commonly used to analyze the flow of information, e.g., in social media, that has recently been applied to neuroscience (Bullmore and Sporns, 2009). Graph analysis relies on the construction of a network in which brain regions serve as nodes, while the connecting edges are defined by the co-activity between the regions which can be determined by the correlation coefficient of cFos expression (Figure 1E). These networks are described in a formal framework, e.g., in terms of modularity–the presence of functionally segregated modules, centrality–the importance of a region in information flow (Figure 1F), or communication efficiency–a measure of how efficiently information can be exchanged between nodes (Newman, 2010). Thus, graph analysis allows inference on which brain regions form higher order neuronal ensembles or meta-ensembles by high degrees of co-activity and what nodes are most influential to control the network. A major advantage of graph analysis is that the extracted network properties can be tested by proper statistical methods. We and others have recently applied this framework to characterize meta-ensembles involved in reward memories (Kimbrough et al., 2020, 2021; Wandres et al., 2021).

Application of graph analysis to cFos-derived networks revealed significant reward-specific differences in network structure, despite the similar ensemble sizes in individual brain regions (Figure 1; Wandres et al., 2021). Specifically, seeking for saccharin engaged a highly modular network with strong correlations between sub-regions of the network (e.g., mPFC), in which the prelimbic and orbitofrontal cortices were the most important for controlling the flow of information. In contrast, the network recruited during alcohol seeking was not modularly organized and showed a weaker but more broadly connected structure instead. This was also reflected in significantly weaker global and local communication efficiencies in the alcohol condition. Notably, in the alcohol meta-ensemble, the most important region for the control of information flow was the anterior insula while the basolateral amygdala also increased in importance (Figure 1G). Given that alcohol represents a multimodal stimulus–smell, taste, caloric content, and pharmacological effects–the involvement of structures mediating the representation of internal states, such as insula and amygdala, in alcohol-associated memory is highly plausible. Interestingly, the importance of the insula and its connection to the amygdala is one of the most consistent findings across AUD studies (Campbell and Lawrence, 2021; Centanni et al., 2021; Flook et al., 2021; Sommer et al., 2022).

Besides the fundamentally different structures of the meta-ensembles in control of alcohol and saccharin seeking, the statistical graph analysis implicates additional important insights about the representation of reward memories that would have gone unnoticed by the common region centered view. First, cFos meta-ensembles are dynamically remapped on demand. The different task-states have been entrained by previous learning. Such task-specific representational networks may serve as priors supporting decision-making in complex environments (Niv, 2019). Second, our inability to differentiate between rewards based on the activity levels of local ensembles suggests that the remapping of cFos meta-ensembles is likely to ultimately rely on synaptic plasticity induced by Hebbian mechanisms or changes in neuronal excitability. Furthermore, the meta-ensemble approach described here resembles the study of functional connectivity in the human brain and may therefor support translation of research findings.

## Conclusion, limitations, and future directions

To conclude, increasing evidence causally links the activity of diverse neuronal ensembles within the reward system to the representation of reward-associated memories and consequently the control of reward-seeking and taking behavior. Network analysis of cFos-derived co-activation patterns across the reward system revealed distinct, clearly separable connectivity states between local ensembles, termed meta-ensembles, in which information transfer was controlled by different brain regions and which could be dynamically recruited upon demand. However, while the meta-ensembles described here may capture some fundamental properties of brain networks, they cannot constitute the entire representation of a specific memory. A postmortem “snap-shot” of a highly dynamic process has inherent limitations, such as the number of observable states, temporal resolution and stability over time. One problem may arise from the specific marker characteristics, such as different expression levels and induction thresholds of cFos in different brain regions or cell types (see e.g., Figure 1D), which may bias the inclusion of brain regions and observable network states. This could potentially be addressed by the use of multiple marker proteins (e.g., Arc, NF-κB). The principle problem, however, exists for all methods. In fMRI, for example, many subcortical areas are seldom examined due to their deep location and small size.

Nevertheless, future studies of reward memories and addiction will need to focus on animal models that properly represent the pathological condition, including a biased choice of alcohol over natural rewards (Meinhardt and Sommer, 2015; Augier et al., 2018; Russo et al., 2018). These studies will benefit from adopting a network perspective by applying systems level approaches and largely unbiased analyses strategies, as recently demonstrated in a large translational AUD research project (Sommer et al., 2022) and the brain-wide investigation of cFos expression in 123 regions from chronically alcohol-drinking mice using light sheet microscopy that elegantly demonstrated decreased modularity of brain networks (Kimbrough et al., 2020). These results are in line with our findings (Wandres et al., 2021) and supported by recent fMRI results from animals (Degiorgis et al., 2022; Pérez-Ramírez et al., 2022) and humans (Bordier et al., 2022; Camchong et al., 2022), Targeting network modules, rather than individual brain regions non-invasively, holds therapeutic potential as demonstrated by a clinical trial in AUD patients using transcranial magnetic stimulation of the mPFC (Harel et al., 2022).

Finally, the better understanding of the dynamics and fluidity of neuronal ensembles will require monitoring and manipulating ensemble activity over extended periods of time and more specifically during behavioral events, e.g., the lever response or reward consumption. Early applications of such technologies are emerging (Brebner et al., 2020; Visser et al., 2020; Domi et al., 2021) and open exciting new possibilities for studying the representation of reward memories and ultimately how to regain control over addictive behaviors (Heinz et al., 2020).

## Author contributions

CK and WS wrote the manuscript. Both authors contributed to the article and approved the submitted version.
